# Are High Levels of Microsatellite Instability and Microsatellite Stability Identical in DNA Mismatch Repair-Deficient Colorectal Cancer Patients?

**DOI:** 10.1155/2023/8370262

**Published:** 2023-03-08

**Authors:** Yan-Yu Qiu, Yi-Xin Zeng, Yong Cheng

**Affiliations:** ^1^Department of Gastrointestinal Surgery, The First Affiliated Hospital of Chongqing Medical University, Chongqing 400016, China; ^2^Chinese Academy of Medical Sciences & Peking Union Medical College Institute of Medicinal Biotechnology, Beijing 100050, China

## Abstract

**Purpose:**

The purpose of the current study was to determine whether there is a difference between high levels of microsatellite instability (MSI-H) and microsatellite stability (MSS) in DNA mismatch repair-deficient (DMMR) colorectal cancer (CRC) patients.

**Methods:**

A total of 452 CRC patients with DMMR from December, 2014, to April, 2021, in our hospital were selected retrospectively. However, only 105 patients underwent Sanger or next-generation-sequencing (NGS) to confirm their microsatellite status. Ultimately, 55 MSI-H patients and 20 MSS patients with intact medical record information were included in this study.

**Results:**

The MSS group was associated with a higher mutation rate in the KRAS gene (*P*=0.011). Meanwhile, MSI-H was related to colon cancer (*P* < 0.01). However, no significant differences in other clinical characteristics were observed between the two groups of patients. There was no significant difference between the MSI-H and MSS groups in terms of overall survival (OS) (*P*=0.398) and disease-free survival (DFS) (*P*=0.307).

**Conclusion:**

The MSI-H status was associated with colon cancer and a lower mutation rate of the KRAS gene in DMMR patients. In CRC-DMMR patients, the MSS group exhibited better OS and DFS than the MSI-H group, although these differences were not statistically significant. Accordingly, in clinical practice, we should not confuse these two types of patients.

## 1. Introduction

Colorectal cancer (CRC) has been identified as the third most frequent cancer and the second leading cause of cancer-related death [1, 2]. There are over 1.8 million new CRC patients and 880,000 deaths from CRC every year [3]. DNA mismatch repair deficiency (DMMR) is one of the mechanisms that lead to genomic instability, which is crucial for the development and progression of CRC [4]. DMMR status is associated with high levels of microsatellite instability (MSI-H). Nevertheless, most CRC patients have a proficient mismatch repair (PMMR) state, which results in microsatellite stability [5–7].

Immune checkpoint inhibitors (ICIs) have been shown to exhibit a durable response and control of disease in advanced CRC [8, 9]. The objective response rate (ORR) and overall survival of patients with MSI-H and CRC-DMMR were dramatically enhanced following the treatment with ICIs [10]. The underlying mechanism was established may be due to the deficiency of DNA mismatch repair increased tumor mutational burden (TMB) and immune cell infiltration [11–14].

It is thought that approximately 15% of CRC patients present with MSI-H, which is caused by the DMMR system, while PMMR confers MSS to the remaining 85% CRC patients [5–7]. Approximately, 10% of the patients are inconsistent in terms of mismatch repair (MMR) and microsatellite status [15]. Therefore, the aim of this study is to investigate whether CRC-DMMR patients with MSI-H and MSS have equivalent outcomes and clinical features.

## 2. Materials and Methods

### 2.1. Inclusion and Exclusion Criteria of Patients

The inclusion criteria of patients were as follows: (1) CRC patients who underwent DNA MMR protein analysis to confirm DMMR status. (2) CRC patients who underwent Sanger or NGS to confirm MSI status. (3) CRC patients with intact medical record information.

The exclusion criteria of patients were as follows: (1) Patients whose MMR status was unknown. (2) Patients whose MSI status was unknown. (3) Patients without intact medical record information.

### 2.2. Microsatellite Instability Analysis

The previous study has described the specific procedure of MSI analysis [16]. The MSI status was evaluated with a five-site panel comprising two mononucleotide sites, BAT-25 and BAT-26, and three dinucleotide sites, D2S123, D5S346, and D17S250. In addition, the panel also contained a sample with a contaminated five nucleotide Penta C site as an internal control. PCR amplification was performed using a 10 *μ*l reaction volume, including 2 × 5 *μ*l polymerase chain reaction (PCR) master mix, 5 × 2 *μ*l 5 primer mix, 0.2 *μ*l AmpliTaq Gold DNA polymerase (5 units/*μ*l), and 5–10 ng DNA templates. PCR was performed on a PE 9600 thermal cycler using the following cycling profiles: 95°C holds for 4 min, 30 cycles at 95°C for 30 s, at 60°C for 30 s, at 72°C for 30 s, and at 60°C for 45 min, and then hold. The use of 30 cycles avoids the formation of shadow peaks. After PCR amplification, the product was detected and analyzed with an ABI 3730 Genetic Analyzer (Applied Biosystems, CA, USA), following the manufacturer's protocol. The data were analyzed with GeneScan Analysis and Genotyper software packages from Applied Biosystems to identify the predominant allele size for each locus. MSI tumor positivity was determined by the number of bases of alleles with corresponding loci and by the internal control index of the tumor samples and their paired normal control samples.

NGS was performed the same way as previously described [17, 18]. In brief, isolated circulating tumor DNA (ctDNA) samples were processed with the KAPA HyperPrep kit (KAPA Biosystems) for library construction. A customized NGS panel targeting 425 cancer-relevant genes was used for hybridization enrichment. Indexed DNA libraries were pooled together to a total amount of 2 *μ*g and subjected to probe-based hybridization using IDT xGen Lockdown reagents (IDT, Coralville, IA) and Dynabeads M-270 (ThermoFisher). The library was quantified using a KAPA Library Quantification kit (KAPA Biosystems) according to the manufacturer's instructions. A Bioanalyzer 2100 (Agilent, USA) was used to determine the fragment size distribution of the final library, which was then sequenced on an Illumina HiSeq4000 NGS (Illumina) platform following the manufacturer's instructions. An expected sequencing depth of 3000× was set for ctDNA samples.

### 2.3. DNA Mismatch Repair Protein Analysis

Immunohistochemical (IHC) analysis of MSH2, MSH6, PMS2, and MLH1 proteins was performed as previously described [16, 19, 20]. Briefly, after the tumor area adjacent to normal mucosa and/or lymphocytic infiltration had been marked, the paraffinized tissue was removed and multiple tissue blocks were prepared. Finally, 4 *μ*m-thick sections were obtained for IHC following standard protocols. The mouse monoclonal antibodies used were anti-MSH2, anti-MSH6, anti-MLH1, and anti-PMS2 (BD Pharmingen, CA, USA).

### 2.4. Statistical Analysis

We used SPSS software (Version 26.0; IBM Corp., New York, USA) to analyze the data. The frequency variables were compared by the chi-squared test (or Fisher's exact test), and the continuous variables were compared by the independent-sample *t* test. The survival curves of OS and DFS were generated using Kaplan–Meier analysis. A log‐rank test was used to test the between‐subgroup differences in survival curves, and *P* ≤ 0.05 was considered statistically significant.

## 3. Results

**Figure 1:**
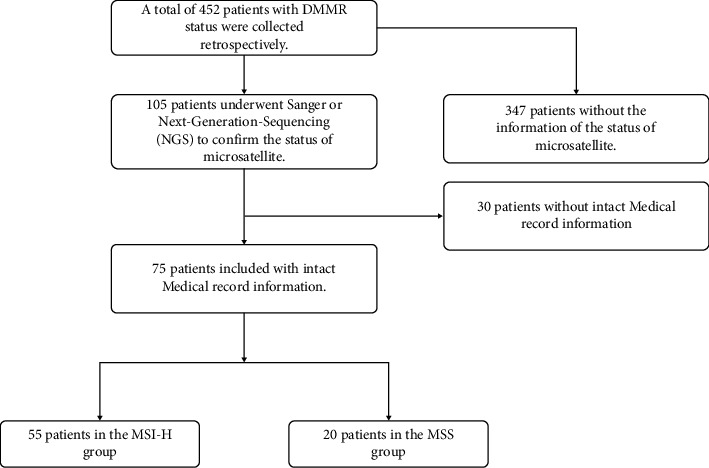
Flowchart of study selection.

We retrospectively collected patients with DMMR status from December, 2014, to April, 2021, in our hospital. There were 452 patients with DMMR status. Of these, 347 who did not undergo Sanger or next-generation sequencing (NGS) were excluded. Thirty patients without complete medical records were excluded. Ultimately, 55 MSI-H patients and 20 MSS patients with complete medical record information were admitted (Figure 1). 37 of the 75 patients were female, with a median age of 62.8 ± 14.0 years. Among these patients, 61 had tumors localized in the colon and 14 in the rectum. In addition, 21 patients were positive for KRAS mutations. The characteristics of these 75 patients are summarized in Table 1.

The MSS group was associated with a higher mutation rate in the *KRAS* gene (*P*=0.011). Meanwhile, the tumor site in MSI-H patients was more likely to be in the colon instead of the rectum (*P* < 0.01). However, no significant differences between the two groups were found with respect to immune-related indicators. The same results were shown for tumor markers and tumor stages. No significant differences were found in other clinical characteristics of the enrolled patients (Table 2).

**Figure 2:**
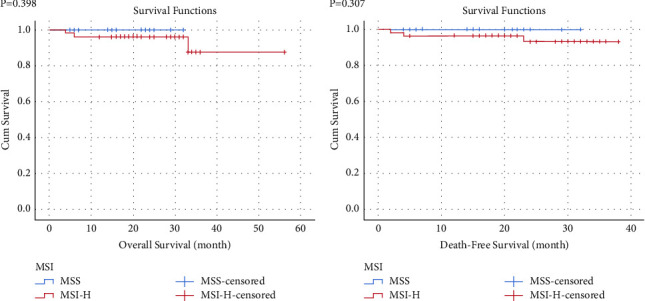
Survival curves of the two groups. (a) *P*=0.398. (b) *P*=0.307.

The survival curves of OS and DFS were analyzed by the Kaplan–Meier analysis. The MSS group was associated with better OS and DFS, but no significant differences were observed (Figure 2).

## 4. Discussion

MSI-H and MSS statuses are often equated in CRC-DMMR. Numerous previous studies have concluded that DMMR causes approximately 15% of CRC patients to present with MSI-H, while the remaining 85% of CRC patients present with MSS caused by the PMMR system [5–7]. Recently, however, a report from Debniak et al. showed that approximately 10% of patients have inconsistent MMR and MSI [15]. There are several explanations for this observation. First, some missense mutations generated by mismatch repair dysfunction proteins may retain antigenicity and be recognized by antibodies and display PMMR status. Second, some MMR proteins may be deficient in activity, but their function may be compensated by other proteins. All these possible causes are speculative, and there may be other possible causes.

We retrospectively collected 75 DMMR patients with intact medical record information who underwent Sanger or NGS to confirm their microsatellite status. The current study was a retrospective study, so we cannot repeat the tests to double-check our results. There were 55 patients in the MSI-H group and 20 patients in the MSS group. Our medical center was late to introduce genetic testing, both in Sanger and NGS. Therefore, fewer patients underwent MSI status testing. There were 30 patients (29 DMMR/MSI-H and only 1 DMMR/MSS) without intact medical record information. Therefore, the percentage of inconsistency between MMR status and MSI status may have some discrepancy with previous data. However, patients selection method was randomised to avoid selection bias.

Most of the patients did not undergo CHT. Four patients underwent neoadjuvant chemotherapy, and 25 patients underwent CHT (22 XELOX and 3 FOLFOX). However, all patients underwent MMR immunohistochemistry and DNA microsatellite testing before chemotherapy (CHT) to prevent chemotherapy from influencing our results. Only 4 patients accepted immunotherapy in the current study because most of the MSI-H patients were in the low TNM stage, and immunotherapy was not popular in our hospital at that time. In addition, the adjuvant CHT regimen was mainly determined by the TNM stage and high-risk factors; therefore, the MSS status did not affect decisions about adjuvant CHT.

Our data suggest that the MSI-H group had a higher colon cancer ratio compared with the MSS group (*P* < 0.01). This finding is consistent with the result of a previous study [21]. This result might suggest that the tumor site is linked with the status of MSI rather than the status of MMR. The current study reveals KRAS mutation is related to MSS in DMMR CRC patients (*P*=0.011). However, the findings of Fujiyoshi et al. are inconsistent with our current results, demonstrating a high concordance between KRAS mutation status and MSI-H status [22]. However, the patients in both studies were in DMMR status. Therefore, we speculate that KRAS mutation has a close relationship with DMMR but not the MSI-H status.

Torshizi Esfahani et al. showed that a difference in OS between the MSS group and the MSI-H group was not evident [23]. However, more studies have shown that MSS is related to a longer OS than MSI-H [24, 25]. The current study suggests that there is no significant difference in OS between the MSS group and the MSI-H group. However, the MSS group in our study was in DMMR status, which suggests that OS might be more tightly related to DMMR status.

Interestingly, the DFS of the MSS group was prolonged, although there was no significant difference in DFS between the MSS group and the MSI-H group in our study. This outcome is contrary to that of Kim et al., who found that CRC patients with MSI-H had better DFS [26]. Therefore, based on the available findings, we speculate that DFS has a closer relationship with DMMR status, because the patients in the MSS group in this study all had a status of DMMR.

The tumor microenvironment (TME) can suppress uncontrolled tumor growth and distant metastasis by activating specific immune responses. Emerging cancer therapies, such as immunotherapy, which can exploit the immune capacity of the TME, are a popular topic [27]. Tumor-infiltrating immune cells, such as cluster of differentiation CD 3+ TILs (tumour-infiltrating lymphocytes, i.e., T cells) and CD8+ TILs (cytotoxic T cells), are important components of the TME. Patients with MSI-H and DMMR have been shown to benefit from immunotherapy [11, 28]. However, the current study found no significant differences with respect to immune-related indicators between the MSI-H group and the MSS group in DMMR patients. The same result was reported in endometrial cancer [29]. Our data suggest that, similar to DFS and OS, the activity of the TME may be closely related to DMMR status, but more experiments are needed.

In the current study, we found that KRAS mutation, OS, DFS, and TME, but not tumor site, were related to DMMR status. These results may assist us in making decisions in cases of inconsistent MMR and MSI statuses. Based on these assumptions, the MMR status may assist in treatment decisions. In turn, the MMR status may be useful in predicting the prognosis of patients more effectively.

To our knowledge, the current study was the first to analyze the difference between the MSS group and the MSI-H group in DMMR patients. We discussed the relationship between MRR status and MSI status, which could help us to estimate the prognosis of patients.

However, there are some limitations to this study: (1) Only 75 patients were enrolled in our study, which was relatively limited; (2) this was a single-center study; (3) we only compared the difference between the MSS group and the MSI-H group in DMMR patients, and the data in PMMR patients were lacking; (4) the median follow-up was only 23 months; (5) this was a retrospective study; and (6) only 4 patients accepted immunotherapy in the current study, and statistical analysis could not be performed Therefore, more multicenter, prospective, large-sample, multiple sets of contrasts, and longer follow-up studies are needed to validate these findings..

In conclusion, the MSS group was associated with a higher mutation rate in the KRAS gene. Meanwhile, MSI-H was related to colon cancer. In terms of OS, DFS, and TME, there was no significant difference between the MSI-H and MSS groups. Considering the results of previous studies, we conclude that DFS, OS, TME, and KRAS mutations are more closely correlated with DMMR status. The tumor site is relevant to the status of MSI. The MMR status can contribute to the design of our treatment plan and predict the prognosis of patients more accurately. In summary, this study suggest that we should not confuse the MSI-H group and the MSS group in DMMR CRC patients.

## Figures and Tables

**Table 1:** Baseline characteristics of CRC patients.

Characteristics	No. 75
MSI-H/MSS	55/20
Sex (male/female)	38/37
Age (mean ± SD) (years)	62.8 ± 14.0
BMI preoperative (mean ± SD) (kg/m^2^)	22.6 ± 2.7
Smoking	27
Drinking	23
Family history	5
ASA (1/2/3)	52/17/6
Tumor site (colon/rectum)	61/14
TNM stage (I/II/III/IV)	13/43/18/1
Lymphatic metastasis	17
KRAS gene	21
BRAF gene	10
Maximum diameter of tumor (mean ± SD) (cm)	5.3 ± 2.4
Recurrence	7/68
CA-199 (mean ± SD) (*μ*/ml)	59.9 ± 263.4
CA-724 (mean ± SD) (*μ*/ml)	7.4 ± 10.1
CEA (mean ± SD) (ng/ml)	12.5 ± 26.8
CD3+ (mean ± SD) (/*μ*l)	1001.3 ± 395.5
CD4+ (mean ± SD) (/*μ*l)	598.9 ± 267.5
CD8+ (mean ± SD) (/*μ*l)	353.4 ± 165

**Table 2:** Correlations between clinic features and MSI status in DMMR CRC patients.

Characteristics	MSI‐H	MSS	Total	*P* value
Total	55	20	75	
Male/female	25/30	13/7	38/37	0.134
Age (mean ± SD) (years)	62.4 ± 14.5	63.8 ± 2.8	62.8 ± 14.0	0.322
BMI preoperative (mean ± SD) (kg/m^2^)	22.5 ± 2.7	22.9 ± 2.5	22.6 ± 2.7	0.262
Smoking (yes/no)	19/36	8/12	27/48	0.663
Drinking (yes/no)	17/38	6/14	23/52	0.940
Family history (yes/no)	4/51	1/19	5/70	0.727
*ASA (full name)*				0.202
1	35	17	52	
2	15	2	1	
3	5	1	6	
Tumor site (colon/rectum)	50/5	11/9	61/14	<0.01^*∗*^
*TNM stage*				0.103
I	7	6	13	
II	34	9	43	
III	14	4	18	
IV	0	1	1	
Lymphatic metastasis (yes/no)	12/43	5/15	17/58	0.771
KRAS gene (wild/mutant)	44/11	10/10	54/21	0.011^∗^
BRAF gene (wild/mutant)	46/9	19/1	65/10	0.200
Maximum diameter of tumor (mean ± SD) (cm)	5.4 ± 2.4	5.1 ± 2.4	5.3 ± 2.4	0.919
Recurrence (yes/no)	5/50	2/18	7/68	0.905
CA-199 (mean ± SD) (*μ*/ml)	73.7 ± 301.2	15.8 ± 14.6	59.9 ± 263.4	0.271
CA-724 (mean ± SD) (*μ*/ml)	9.6 ± 11.6	2.8 ± 2.6	7.4 ± 10.1	0.139
CEA (mean ± SD) (ng/ml)	11.6 ± 23.4	15.3 ± 36.4	12.5 ± 26.8	0.415
CD3+ (mean ± SD) (/*μ*l)	973.5 ± 412.6	1069.7 ± 353.7	1001.3 ± 395.5	0.356
CD4+ (mean ± SD) (/*μ*l)	579.4 ± 287.7	647.2 ± 210.5	598.9 ± 267.5	0.176
CD8+ (mean ± SD) (/*μ*l)	348.2 ± 163.4	366.2 ± 174.7	353.4 ± 165.2	0.97

## Data Availability

The data used to support the findings of this study are included in the article.
